# Effects of Dietary Methionine Restriction on Cognition in Mice

**DOI:** 10.3390/nu15234950

**Published:** 2023-11-29

**Authors:** Hannah Lail, Angela M. Mabb, Marise B. Parent, Filipe Pinheiro, Desiree Wanders

**Affiliations:** 1 Department of Nutrition, Georgia State University, 140 Decatur St SE, Atlanta, GA 30303, USA; hland2@student.gsu.edu (H.L.); jpinheiro@gsu.edu (F.P.); 2 Department of Chemistry, Georgia State University, 100 Piedmont Ave., Atlanta, GA 30303, USA; 3 Neuroscience Institute, Georgia State University, 100 Piedmont Ave., Atlanta, GA 30302, USA; amabb@gsu.edu (A.M.M.); mbparent@gsu.edu (M.B.P.); 4 Center for Behavioral Neuroscience, Georgia State University, Atlanta, GA 30302, USA; 5 Department of Psychology, Georgia State University, 140 Decatur St SE, Atlanta, GA 30303, USA; 6 Faculty of Medicine, University of Porto, Alameda Prof. Hernâni Monteiro, 4200-319 Porto, Portugal

**Keywords:** age, Alzheimer’s disease, behavior, brain, cognition, female, high-fat diet, methionine restriction, obesity, sex

## Abstract

Dietary restriction of the essential amino acid, methionine, has been shown to induce unique metabolic protection. The peripheral benefits of methionine restriction (MR) are well established and include improvements in metabolic, energy, inflammatory, and lifespan parameters in preclinical models. These benefits all occur despite MR increasing energy intake, making MR an attractive dietary intervention for the prevention or reversal of many metabolic and chronic conditions. New and emerging evidence suggests that MR also benefits the brain and promotes cognitive health. Despite widespread interest in MR over the past few decades, many findings are limited in scope, and gaps remain in our understanding of its comprehensive effects on the brain and cognition. This review details the current literature investigating the impact of MR on cognition in various mouse models, highlights some of the key mechanisms responsible for its cognitive benefits, and identifies gaps that should be addressed in MR research moving forward. Overall findings indicate that in animal models, MR is associated with protection against obesity-, age-, and Alzheimer’s disease-induced impairments in learning and memory that depend on different brain regions, including the prefrontal cortex, amygdala, and hippocampus. These benefits are likely mediated by increases in fibroblast growth factor 21, alterations in methionine metabolism pathways, reductions in neuroinflammation and central oxidative stress, and potentially alterations in the gut microbiome, mitochondrial function, and synaptic plasticity.

## 1. Introduction

Methionine is one of nine essential amino acids that cannot be synthesized by the body and must be obtained through diet to sustain life in all human and animal species [1]. Though the complete elimination of essential amino acids from the diet can have harmful [2], even lethal [3] effects, their controlled restriction can induce favorable metabolic benefits [4]. In particular, the restriction of dietary methionine by about 80% of *ad libitum* intake has been shown to induce unique metabolic protection in adult rodent preclinical studies [5]. The benefits of dietary methionine restriction (MR) are well established and include improvements in body weight [6,7], adiposity [7], energy expenditure [8], insulin sensitivity [6,9], glucose tolerance [6,9], inflammation [10,11], oxidative stress [12], dyslipidemia [6], and extended lifespan [13,14]. These metabolic benefits, which mirror the well-known benefits of calorie restriction, occur despite MR increasing energy intake [15]. This makes MR an attractive dietary intervention for the prevention or reversal of conditions such as obesity, type 2 diabetes, cardiovascular disease, and potentially autoimmune disorders. While most of MR’s known benefits pertain to peripheral systems, new and emerging evidence suggests that MR also benefits the brain and promotes cognitive health [16].

**Figure 1:**
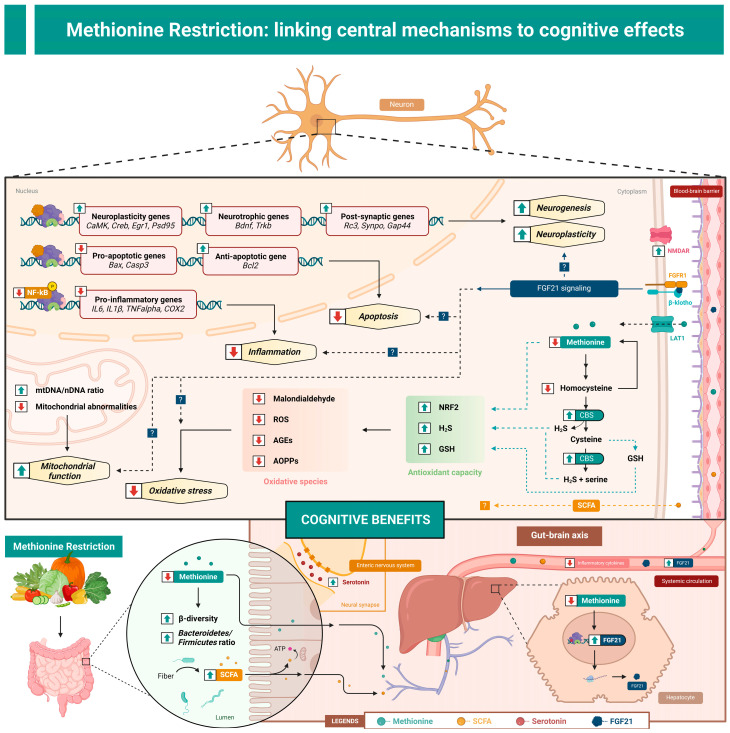
The neurometabolic link between cellular mechanisms and cognitive outcomes in dietary methionine restriction (MR). MR improves cognition via simultaneous improvements in neurogenesis, neuroplasticity, mitochondrial function, inflammation, oxidative stress, and apoptosis. These effects are potentially mediated by direct effects of methionine restriction in the brain and indirect effects via the increased production of fibroblast growth factor 21 in the liver and short-chain fatty acids in the gut. *Abbreviations*. AGEs, advanced glycation end products; AOPPs, advanced oxidation protein products; CBS, cystathionine-β-synthase; FGF21, fibroblast growth factor 21; FGFR1, fibroblast growth factor receptor 1; GSH, glutathione; LAT1, L-type amino acid transporter 1 NMDAR, N-methyl-D-aspartate receptor; NRF2, nuclear factor erythroid 2-related factor 2. Created with Biorender.com.

Currently, the exact cellular mechanisms mediating MR’s central benefits are widespread yet underdeveloped. This may be due to the fact that methionine serves many functions throughout the brain and body making it difficult to narrow down exactly how and where MR is acting to improve cognition. That said, a few key hypotheses seem to be most prevalent within the literature and will therefore be discussed herein. Indeed, MR’s cognitive benefits are likely mediated by alterations in numerous biological systems including methionine metabolism, reductions in neuroinflammation and central oxidative stress, increases in fibroblast growth factor 21 (FGF21), and potentially alterations in the gut microbiome, mitochondrial function, and synaptic plasticity, discussed in detail later (Figure 1).

Despite widespread interest in MR over the past few decades, many findings are limited in scope, and to this day, gaps remain in our understanding of its comprehensive effects on the brain and cognition. This review details the current literature [3,17,18,19,20,21,22,23] investigating the impact of MR on cognition in various mouse models, highlights some of the key mechanisms responsible for its cognitive benefits, and identifies gaps that should be addressed in MR research moving forward.

### 1.1. MR: Working Memory

Working memory is an executive function most closely associated with the prefrontal cortex [24,25,26]. However, working memory also relies heavily on the integrity of the hippocampus [27,28,29]. This specific memory skill set is essential for the ability to plan, implement self-control, follow directions, and focus. Unlike short-term or long-term memory, working memory is not consolidated or made permanent in the brain. Rather, it is discarded when the information is no longer needed. Therefore, working memory enables the brain to acutely hold incoming information, making it essential for more challenging tasks such as learning [30]. The most common tests used to measure working memory *in vivo* are those that assess an animal’s ability to perform trial-unique procedures that do not involve memorization. There are many different cognitive tests that can be used to assess spatial working memory. Some of the most commonly used tests are the Y-maze [31], T-maze [32], and plus maze [33]. However, each of the MR studies reviewed herein assessed spontaneous alternation in the Y-maze [17,18,22,23,28]. In this task, animals explore an elevated maze that contains three equally spaced arms for a specified amount of time. The sequence of entries and the number of entries to each arm are recorded and an alternation index (percent spontaneous alternation) [28] is calculated as an indicator of spatial working memory. In general, healthy animals with intact working memory should remember which arms have been most recently visited and will show a greater tendency to enter the less recently visited arms. This is reflected by a high percentage spontaneous alternation score that is well above a 50% chance level [31].

Age is associated with a natural decline in working memory. In humans, studies have evaluated whether age-related decline in working memory differ between the types of information being tested. Indeed, visuospatial working memory is one of the first cognitive processes to diminish with age [34], making the spontaneous alternation Y-maze an especially relevant behavioral assessment to study the impact of aging. For example, in one study, the Y-maze was used to measure animals’ percent spontaneous alternation to determine whether a 3-month MR diet could attenuate age-related impairments in the working memory of male and female mice aged 5, 15, and 18 months [17]. As expected, the percent spontaneous alternation of 18-month-old mice was significantly lower than that of 5-month-old mice, indicating an age-induced decline in spatial working memory [17]. Notably, MR improved the percent spontaneous alternation of 15-month-old males, and 18-month-old males and females compared to their control-fed counterparts [17].

As a further demonstration of the benefits of MR on working memory, researchers used the D-galactose (d-Gal) model, which induces an age-like phenotype in rodents. D-Gal-induced aging increases reactive oxygen species (ROS) and leads to the deterioration of cognitive and motor skills, similar to the physiological and behavioral changes that occur in natural aging [35]. In a male mouse model of d-Gal-induced aging, just 8 weeks of MR was sufficient to restore spontaneous alternation in Y-maze to that of controls [18]. These data further support that MR can be used to improve age-related decreases in working memory.

In addition to age, poor diet is known to have a profound effect on cognitive health. Specifically, diets high in animal-derived saturated fat, or high-fat diets (HFD), impair cognition independent of body weight- [36] or age-related [37,38,39] decline. MR is a dietary intervention shown to protect against the harmful effects of HFD in preclinical models [40]. It is then unsurprising that MR also improves HFD-related impairments in working memory. In this well-powered study, 10-week-old male mice were fed a control or HFD for 4 weeks to induce obesity [23]. Mice were then switched to either control or HFD versions of normal methionine (0.86% of the diet by weight) or MR (0.17% of the diet by weight) diets for the remaining 8 weeks (*n* = 42/group) [23]. By week 4, mice on the HFD weighed significantly more than controls and showed signs of insulin resistance. Importantly, MR reversed this effect within 2 weeks of switching to the high-fat MR diet [23]. After 8 weeks on their respective diets, the mice underwent Y-maze testing and again MR was shown to significantly increase spontaneous alternation compared to the HFD group [23].

While low methionine intake improves learning and memory, excessive methionine intake has the opposite effect [41]. In male mice, high-methionine intake (2.58% of the diet by weight) impaired working memory with significant reductions in spontaneous alternation within 60 days of diet initiation compared to normal-methionine intake (0.86% of the diet by weight) or MR (0.17% of the diet by weight), assessed via Y-maze [22]. However, unlike the other studies reported herein, MR (0.17% of the diet by weight) did not improve working memory compared to normal-methionine intake (0.86% of the diet by weight) [22]. This may be because young adult mice (aged 9 weeks) were used for these studies. These young, healthy mice would not be expected to exhibit any cognitive deficits in this simple task, so it is not surprising that MR failed to improve spontaneous alternation in this population. In summary, MR is a dietary intervention that can be used to improve age- and diet-related deficits in working memory as shown by various studies assessing spontaneous alternation in the Y-maze [17,18,22,23].

### 1.2. MR: Episodic Learning and Memory

Episodic memory is a subclass of declarative memory that involves the conscious recollection of past personal experiences and their context including who, what, when, and where [42]. Episodic memory is an essential function that relies on the hippocampus, parahippocampal regions, and the prefrontal cortex [43]. One of the most common behavioral tests used to assess episodic memory in mice, specifically recognition memory, is the novel object recognition test (NORT). This assessment is based on the spontaneous tendency for rodents to spend more time with a new or novel object compared to a familiar one. NORT is conducted in an open field arena. During the habituation day(s), animals are allowed to explore an empty arena to become familiarized with the testing environment. Following habituation, the animals are placed back into the arena, this time containing two identical objects. On testing day(s), the animals are allowed to explore the arena in the presence of one familiar object from the previous trial and one novel object to test short-term or long-term recognition memory, depending on the duration between exposures. The time spent exploring each object and a discrimination index is calculated. A positive recognition index signifies a preference toward the novel object and a high recognition score is indicative of strong recognition memory.

For years, this behavioral test has been used to detect changes in cognitive ability in response to different diets and conditions including MR [20], HFDs [39], obesity [44], and age [45]. As mentioned previously, HFDs induce cognitive decline and affect episodic memory after as little as one day on the diet [39]. That said, MR has primarily been investigated for its restorative rather than preventative effect on obesity- and age-related cognitive impairments, leaving a gap in our knowledge of MR as a proactive dietary intervention that can protect against or even prevent acute HFD-induced impairments in learning and memory. Even so, MR clearly has profound effects on recognition memory supported by gains in novel object exploration time and improvements in the discrimination indexes of obese [3,20,21,22] and aged [17,18] males, detailed below.

In a young model of diet-induced obesity, 5-week-old male mice were placed on HFDs for 10 weeks to induce obesity [20]. Control mice continued a control diet for an additional 16 weeks. To determine MR’s restorative effects, the HFD group was split into two treatment groups. The first group continued the HFD, and the second group was switched to a high-fat MR (0.17% of the diet by weight) [20] diet for 16 weeks. Long-term HFD feeding impaired animal performance in NORT, shown by significant reductions in the time spent with the novel object versus the familiar object, and significant reductions in the discrimination index score compared to controls [20]. MR reversed this effect and significantly increased both the time spent with the novel object and the novel object discrimination index [20]. In a similar fashion, another study found a significant increase in the time spent with the novel object of 9-week-old male mice on MR (0.17% of the diet by weight) compared to mice on a high-methionine diet (2.58% of the diet by weight) [22]. Similar to the previous outcomes reported in this study [22], there was no statistical difference between the NORT performance of MR (0.17% of the diet by weight) and normal-methionine intake (0.86% of the diet by weight) groups [22]. That said, both groups showed significant improvements in discrimination indexes compared to the high-methionine group (2.58% of the diet by weight), indicating that in general, lower methionine intake contributes to the preservation of cognitive health [22].

These studies present some interesting findings for MR and cognition; however, young animals are typically healthier and respond more efficiently to metabolic stressors than older animals [46]. In addition, young mice rarely present with cognitive deficits, making it difficult to investigate MR’s comprehensive effects on cognition. Thus, MR’s neurocognitive benefits have also been investigated in mouse models of middle-aged obesity [3,21]. Among the many adverse health outcomes associated with mid-life obesity is the development of mild cognitive impairments [47,48]. Moreover, excessive fat intake exacerbates cognitive decline and is associated with the development of neurodegenerative diseases such as Alzheimer’s disease (AD) [49,50] later in life. Therefore, one study aimed to determine whether MR could protect against HFD-induced obesity-related cognitive impairments during middle age [3]. As predicted, middle-aged, 8-month-old, male mice consuming the HFD had impairments in recognition memory shown by significant reductions in the time spent with the novel object and a lower discrimination index score in NORT compared to controls, while MR provided complete protection against this effect [3]. This study is not alone in its findings as another evaluation, using an almost identical middle-aged male mouse model, found that MR produced similar improvements in NORT performance independent of body weight or fat intake [21].

The final MR study that assessed NORT used naturally aged male and female mice to determine whether MR could improve age-related impairments in episodic memory [17]. During their initial assessment, using males only, MR significantly improved the NORT performance of 15- and 18-month-old male mice [17]. However, upon additional testing aimed to examine the effect of sex, MR failed to alter the NORT performance of 18-month-old males or females in a side-by-side comparison, despite trending to do so [17]. As to why the findings in males were inconsistent, the second behavioral assessment was not sufficiently powered with only 6 to 7 mice per group, compared to 8 to 10 mice per group during the initial assay [51]. Female groups were also inadequately powered, with only seven mice per group, and so despite MR trending towards improving recognition memory in both males and females, these effects did not reach statistical significance [17]. Together these findings implicate MR as a dietary strategy capable of restoring recognition memory under conditions of obesity- and age-related deficits.

### 1.3. MR: Spatial Reference Learning and Memory

Spatial reference memory refers to the storage and retrieval of information that is related to an object’s location and orientation, such as planning, taking, or remembering a path to a specific location and recalling where and how that location exists within space [52]. It is a critical aspect of cognition in humans and animals alike and is thought to be one of the primary functions of the hippocampus [53]. Considering that spatial reference memory is long term and therefore consolidated in the brain, tests used to measure spatial cognition are repetitive in nature. Many different tests can be used to assess spatial learning and memory [54], but two of the most commonly used in animal research are the Barnes maze (BM) and the Morris water maze (MWM) [55]. The BM is composed of an elevated steel table rimmed with twenty equally spaced holes. A target hole is selected by the researcher to which an “escape box” is tethered underneath. Over the course of several days, the animals explore the table and are tested for their ability to locate and escape to the target hole location. By contrast, the MWM consists of a pool equipped with an elevated “escape platform”. Over the course of testing day(s), animals are assessed for their ability to swim and escape to the target platform. Both tests measure an animal’s ability to learn and recall the position of an escape box or escape platform within a three-dimensional space. In both, the animals are habituated, tested, and oftentimes probed for cognitive flexibility in the presence of spatial cues [55]. Animals that enter the target zone first, spend most of their time in the target region or locate and/or escape to the target box or platform are considered to have superior spatial learning and memory.

To date, only a single study has used BM to assess MR’s impact on spatial cognition [17]. To avoid one of the major setbacks of the MWM (i.e., the stress that swimming causes mice), BM was used to assess 5- and 18-month-old male and female mice, while the MWM was implemented for 5- and 15-month-old male mice [17]. For ease of comparison, the team used identical testing schedules for both including a place navigation test, in which the animal was blinded to the target hole/platform location that was followed by a probe trial test where the platform/hole was removed. In the BM place navigation test used to evaluate spatial learning ability, MR improved the escape latency of 18-month-old mice, regardless of sex [17]. Moreover, during the probe trial test of BM measuring spatial memory, MR increased the number of entries near the target box of 18-month-old males only [17]. In comparison, during the MWM place navigation test, MR improved the escape latency of 15-month-old male mice [17]. Whereas during the MMW probe trial test, MR enhanced the number of platform crossings and the percentage of distance traveled in the target quadrant of 15-month-old males [17]. Together these experiments demonstrate that MR can ameliorate age-induced cognitive impairment in spatial reference learning and memory.

In comparison, each of the eight primary research articles included in this review [3,17,18,19,20,21,22,23] used the MWM, making it the most replicated test by far. Moreover, almost all studies reported improvements in one cognitive measure or another. For example, in one study, 8-month-old male mice were placed on control, HFD, or high-fat MR diets for 25 weeks [3]. MR not only significantly decreased the escape latency, but also increased the number of entries to the target quadrant and target platform, increased the time spent in the target quadrant, and increased the total distance traveled in the target quadrant compared to mice fed the HFD [3]. Therefore, MR can protect against HFD-induced impairments in the long-term spatial cognition of middle-aged males.

While an 80% reduction in dietary methionine is the most common application of MR in animal research and has been shown to be the most effective throughout the periphery [56], 60% MR may also improve spatial cognition. For example, both a 60% reduction and an 80% reduction in dietary methionine improved animal performance in MWM [21]. In this study, male mice were placed on a low-fat diet (4% fat-derived calories), a medium-fat diet (12% fat-derived calories), or a HFD (22% fat-derived calories) containing different levels of MR (0%, 60%, 80%) for 16 weeks [21]. Both 60% and 80% MR significantly increased the number of entries to the target platform and the platform quadrant [21]. However, only an 80% reduction of dietary methionine improved the remaining MWM parameters, including the time and distance traveled in the target platform quadrant consistently across all dietary groups [21]. In a separate study, high-methionine intake (2.58% of the diet by weight) significantly impaired escape latency compared to normal-methionine intake (0.86% of the diet by weight) and MR (0.17% of the diet by weight), [22] yet again providing evidence that lower methionine intake is conducive to better cognitive health [22].

Until recently, no detrimental effects of MR on cognition had been reported. However, a 2023 study found that MR impaired the spatial memory of wild-type mice [19]. The goal of this study was to investigate the neuroprotective effects of MR on mild cognitive impairment using wild-type and an APP/PS1 mouse model of AD [19]. Though the mice were only 4 months of age, this transgenic model quickly developed AD as shown by significant increases in amyloid-β accumulation in the cortex and hippocampus [19]. After 16 weeks, MR unexpectedly impaired the escape latency of wild-type males, but not wild-type females, on day 5 of the working memory navigation test, suggestive of impaired spatial learning [19]. Furthermore, during the probe trial phase of this test, MR decreased the time spent in the target quadrant of wild-type males and females compared to controls, again indicating a decline in spatial memory [19]. Nonetheless, MR significantly increased the time spent in the target quadrant of male mice with AD [19]. The reason why MR impaired wild-type performance and why it preferentially improved cognition in males and not females with AD remains unclear [19]. In summary, all eight studies investigating the effects of MR on cognition [3,17,18,19,20,21,22,23] have examined its effect on spatial reference cognition. Despite some variability in the outcomes, MR has been generally shown to reverse age-, obesity-, and AD-induced deficits in spatial learning and memory, specifically in the MWM. Going forward it will be important to identify whether MR consistently and reliably induces counterintuitive effects on spatial cognition in healthy models, and if this study is an anomaly [19].

### 1.4. MR: Anxiety-like Behaviors

In mice, anxiety manifests as increased vigilance, prolonged periods of freezing and/or hypoactivity, tachycardia, and suppressed food consumption that are used together to discern an animal’s “anxiety-like behavior”. This behavioral response is attributed to the activation of the amygdala [57] and hippocampus [58] and can be easily quantified by an animal’s behavior in an elevated plus maze (EPM). The EPM consists of a raised plus-shaped maze with two closed arms (with walls), and two open arms (without walls), positioned opposite each other with an exposed region in the center. The maze is typically brightly illuminated to induce a mild stress response [59]. As an animal explores the maze, their behavior is recorded. Their anxiety-like behavior is scored as the number or percent of arm entries, and/or time or percent time spent in the open arms. Animals with less anxiety are expected to spend a greater amount of time in and have more entries into the open arms.

As mentioned previously, d-Gal-induced aging increases oxidative stress and promotes cognitive decline [18]. However, the effects of chronic d-Gal administration are not limited to its detrimental effects on the brain. In fact, d-Gal has been shown to alter the gut microbiome, thought to be partially responsible for its central pro-inflammatory effects [18,60], and is associated with the development of behavioral pathologies, such as depression and anxiety [61]. In a model of d-Gal-induced aging, 9-week-old male mice were fed either a control diet, administered d-Gal on a control diet, or administered d-Gal on an MR diet for 8 weeks [18]. At baseline, all groups exhibited similar activity levels with no reported differences in the total distance traveled, time spent in the center, distance traveled in the center, or entries to the center of EPM [18]. In comparison, during testing, mice on the MR diet displayed reductions in anxiety-like behaviors shown by significant increases in the time spent in the open arms, a higher number of entries into the open arms, and higher percent time and percent entries to the open arms compared to the d-Gal group [18]. MR even trended to reduce anxiety levels compared to the control mice not administered d-Gal [18]. Therefore, MR can also improve d-Gal-induced anxiety-like behaviors in a relatively stressful environment, such as the EPM.

Three other studies using MR have found similar beneficial reductions in anxiety using the EPM. Young male mice following a high-fat MR diet for 16 weeks starting at 5 weeks of age had significantly reduced anxiety-like behavior in the EPM compared to HFD-fed mice [20]. However, in this case, MR did not improve anxiety beyond that of controls [20]. Under conditions of varying fat intake, 80% MR was most effective at reducing anxiety measures in EPM, though 60% MR also increased the time spent in the open arms [21]. Lastly, compared to high-methionine intake (2.58% of the diet by weight), both normal-methionine intake (0.86% of the diet by weight) and MR (0.17% of the diet by weight) improved anxiety-like behaviors in EPM, with 80% MR (0.17% of the diet by weight) producing the greatest effects [22].

As an alternative approach to the EPM, the open field test (OFT) can also be used to assess anxiety. The OFT is used to measure general exploratory behavior, activity levels, and anxiety-like behaviors in rodents [62]. The success of this test depends on the hippocampus, amygdala, as well as various cortical and motor regions of the brain [63]. The OFT is simple in its design consisting of a single enclosed arena with walls high enough to prevent an animal from escaping. The size and shape may differ between studies but must be adequate to provoke a feeling of openness when the animal is in the center of the arena. To test, animals are placed in the arena and their activity is tracked for a specified amount of time. Exploration activity and anxiety-like behaviors are scored based on the total distance traveled, average speed, rearing movements, total distance traveled in the center region, time spent in the center region, number of entries to the center region, and resting time. Animals with less anxiety spend more time in the center of the arena and have more activity and less resting time.

Using the OFT, one study examined the protective effects of MR on exploratory activity and anxiety in naturally aged mice and found that increased age did not affect the exploratory behavior of the mice [17]. Despite this, MR decreased the resting time of 18-month-old males, but not females, and trended toward increasing the time males spent in the central region [17]. Moreover, d-Gal-accelerated aging failed to alter exploration activity and anxiety-like behaviors compared to controls [18]. Therefore, MR was unable to produce any protective effects in this model [18].

The protective effect of MR on exploration and anxiety-related behavior of obese mice appears to be more promising than the effects seen in aged models. Compared to controls, HFD-induced obesity significantly reduced the total distance traveled, distance traveled in the center, time spent in the center, and the number of entries to the center in the OFT of male mice [20], indicative of increased anxiety. However, MR restored these parameters in the high-fat MR group to values comparable to mice on the control diet [20]. In another study, both 60% and 80% MR increased the total distance traveled, distance traveled in the center, time spent in the center, and the number of entries to the center of HFD-fed male mice in the OFT [21]. That said, 80% MR produced the most consistent and greatest improvements under conditions of varying fat intake, including controls [21]. It is important to consider that these beneficial effects on performance could be indirectly attributed to MR’s effect on body weight reduction. However, 80% MR improved neuromotor function independent of fat intake using tests measuring grip strength and pole climbing ability [21]. These findings suggest that MR can increase general exploration activity and improve anxiety independent of reductions in body weight.

On the contrary, OFT performances did not differ between MR (0.17% of the diet by weight), normal-methionine (0.86% of the diet by weight), or high-methionine intake (2.58% of the diet by weight), despite high methionine inducing notable increases in anxiety-like behavior in the EPM in the same study [22]. Taken together, these findings demonstrate that MR’s beneficial effects on anxiety depend heavily on the origin of the impairment. For example, MR was most effective for the restoration of obesity-induced [20,21] and high-methionine-induced [22] exploration deficits and anxiety than age-related deficits [17,18], specifically in males. Taking into consideration the simplicity of this test, future MR studies should incorporate the OFT into their behavioral analyses to determine whether MR can protect against AD-induced anxiety. Perhaps the employment of additional behavioral approaches such as the light–dark box assays and social avoidance tasks would help to better describe MR’s impact on anxiety-like behavior [64] and resolve some of the current inconsistencies between the EPM and OFT results.

### 1.5. MR: Linking Cognitive Effects to Mechanisms

All articles detailed in this review examined the brain for physiological changes to establish a neurometabolic link between their *in vivo* models and cognitive outcomes (Figure 1), some of which have already been reviewed [16]. The most common pathways investigated thus far are those involving neuroinflammation and central oxidative stress. This is logical considering that inflammation [65] and oxidative stress [66] are known to promote various metabolic and morphological disturbances in the brain stimulating brain atrophy and eventually resulting in the development of cognitive impairments. The most well studied so far are the nuclear factor kappa-light-chain-enhancer of activated B cells (NF-κB) pathway and radical-induced oxidative damage. For example, six different studies reported MR-related reductions in protein or the gene expression of NF-κB as well as its downstream inflammatory products including tumor necrosis factor alpha, interleukins (IL-6) and (IL-1β), cyclooxygenase 2, either in circulation or in the brain [17,18,20,21,22,23]. Comparatively, five studies reported decreases in malondialdehyde [3,17,18,19,21], four in ROS [3,18,21,22], two in advanced oxidation protein products [18,20], and one in advanced glycation end products [18], after anywhere from 8 and 25 weeks of MR feeding. Mechanistically, these reductions are attributed to MR’s ability to upregulate anti-inflammatory transcription factors such as nuclear factor erythroid 2-related factor 2 [3,18,21], and increase antioxidants including glutathione (GSH) [18,19,22] and hydrogen disulfide (H_2_S) [18,19,20,21], resulting in higher total antioxidant capacity [3,18]. These studies support the use of dietary MR to reduce neuroinflammation and central oxidative damage, which are associated with better cognitive outcomes.

Aging is characterized by a natural progression in mitochondrial dysfunction that occurs due to an accumulation of mitochondrial DNA (mtDNA) mutations and increased ROS in the brain [67]. Mitochondria are an invaluable component of memory [68]. First, they are positioned locally within dendrites and axons and provide neighboring synapses with on-demand energy. Furthermore, in addition to serving as the main energy source for dendritic protein translation, they play an important role in maintaining membrane potentials, the shuttling and release of neurotransmitters, and are essential for synaptic plasticity [69,70]. Two of the MR studies that used age-related models found that aging diminished mitochondrial growth and led to increases in mitochondrial abnormalities characterized by vacuole cavitation, mitochondria shrinkage, and a reduction in mitochondrial cristae [17,19]. Impressively, MR restored mtDNA/nDNA ratios and decreased mitochondria abnormalities and damage in the cortex of male mice [17,19]. Moreover, MR was also found to protect against mitochondrial abnormalities in aged females [17]. These functional changes align with improvements in cognitive performances. Together, these findings implicate mitochondria as an important molecular target for improving age-related impairments in learning and memory. More studies must be performed to examine whether MR can be used to enhance mitochondrial function in additional models including HFD-obesity- and AD-induced mitochondrial dysfunction.

The metabolism of methionine can be broken down into three distinct pathways including the methionine cycle, the transsulfuration pathway, and the salvage cycle [71]. A number of MR studies described herein examined parts of the methionine cycle, including the accumulation of homocysteine (Hcy) or its metabolites in the brain. Hcy is an amino acid involved in the synthesis and metabolism of methionine to proteins in the salvage cycle, or to cysteine, GSH, and H_2_S, in the transsulfuration pathway. Under normal conditions, Hcy remains relatively low and its turnover by the endogenous enzyme cystathionine-β-synthase (CBS) to downstream antioxidant molecules GSH and H_2_S is high. However, during states of metabolic stress such as obesity and disease, including AD, there is an accumulation of Hcy in the brain, which is at least partially responsible for detrimental effects on cognition [72]. Importantly, MR was shown to decrease Hcy levels in the hippocampus of HFD-induced obese mice [20], while increasing CBS and/or H_2_S in multiple brain regions, including the cortex [19,20,21] and hippocampus [18,20]. Alterations in H_2_S were also proposed as the potential mechanism underlying the neuroprotective effects of lower methionine intake as compared to high-methionine intake [22], although it was not directly assessed in this work. Over the years, a multitude of studies assessing MR in tissues other than the brain have found similar benefits stemming primarily from MR’s ability to shift methionine metabolism towards transsulfuration and increase H_2_S [73,74,75,76,77,78], making H_2_S a likely essential component of MR’s cognitive benefits.

Another mechanism repeatedly explored in the MR studies, aside from central inflammation and oxidative stress, is the role of the gut microbiota. Emerging research suggests strong links between the gut and cognitive health, making the gut–brain axis a novel target to enhance cognition. In particular, there is a strong emphasis on a bottom-up immune response, neurotransmitter production and release, and the diversity of the microbiome itself. Indeed, three out of the four [17,18,22,23] MR studies that assessed spontaneous alternation in the Y-maze found that in addition to enhancing spatial working memory, MR improved β-diversity, restored the *Bacteroidetes/Firmicutes* ratio [18,22,23], increased short-chain fatty acid-producing bacteria [22,23], and upregulated gut-derived serotonin [21,22], all found to contribute to better cognitive performance [18,21,22,23].

Many of the metabolic benefits produced by MR can been attributed to a mechanism that involves the upregulation of FGF21, a peptide that is predominantly synthesized by the liver [7,8,79,80]. Interestingly, one article found that MR paradoxically decreased the gene expression of the FGF21 receptor and essential coreceptor (*Fgf1r* and *Klb*) in the hippocampus of aged, but not young, male mice [17]. In addition, they found that the extent of MR-stimulated increases in FGF21 protein expression in the liver, brain, and blood were blunted by increased age [17]. Despite these findings, MR was found to depend upon FGF21 to improve the cognition of aged males using a global viral knockdown model [17]. Recent investigations using FGF21 administration and tissue-specific knockdown models have highlighted sex differences in the essential role of FGF21 in cognition [81]. For example, FGF21 administration had no effect on female spatial memory formation [81]. This could perhaps explain the sex-dependent effects reported in this study [17].

Other molecular changes to the brain have also been implicated in MR’s cognitive benefits. For example, two different studies found that MR protected against obesity-induced anxiety in OFT and EPM [20,21]. Both also reported MR-induced increases in the gene expression of markers related to synaptic plasticity and neuronal growth in the hippocampus [20,21]. Altogether, MR consistently increased N-methyl-D-aspartate receptor subunits including *Nr2a* and *Nr2b*. MR also increased plasticity-related genes *Cam kinase*, *Creb*, *Egr1*, *Psd95*, neurotrophic factors *Bdnf* and *Trkb*, and postsynaptic genes *Rc3*, *Synpo*, and *Gap43* across multiple studies [18,20,21]. MR also worked in a parallel fashion to inhibit neuronal cell death via decreases in apoptotic markers in the brains of mice. For instance, MR reduced apoptosis-promoting genes, *Bax* and *Casp3*, and increased *Bcl2* in the hippocampus of young obese [22] and middle-aged obese male mice [3]. It is now well established that MR can improve hippocampal health by promoting anti-inflammatory and antioxidant pathways, enhancing gene markers of hippocampal plasticity, and improving factors related to neuron growth and survival. Having said that, gene expression analysis only provides indirect evidence of MR’s mechanism for memory improvement. Gene expression is not sufficient to report changes in synaptic plasticity. Therefore, future studies should implement more definitive techniques such as the use of electrophysiology [82] to investigate whether MR is improving cognition at the synaptic level via alterations in synaptic plasticity (i.e., long-term potentiation and long-term depression).

### 1.6. Future Directions

Consolidating the current literature on MR makes it clear that research into MR’s impact on female cognition is lacking. Despite several sex-dependent results having been reported [17,19], few studies have investigated, and none have identified, exactly how and why MR may not be as effective in females as in males. Moreover, females are known to respond differently to dietary challenges, particularly MR [83], making it likely that their cognitive responses will also vary. Notably, many of the behavioral tests developed to assess cognition in rodents display bias toward male outcomes [84,85]. For instance, some early findings suggest that males’ inherent interest in the novel object in NORT may be greater than that of females’ [84], potentially resulting in an inaccurate interpretation of MR’s effect on female recognition memory. Moreover, females are found to implement different strategies in the MWM [85] and are even reported to have higher baseline activity than males [86], again potentially confounding behavior findings. So far, only two studies, out of the eight MR studies reported herein, included females in their neural and cognitive analyses. Both studies used age-related models (naturally aged or transgenic AD) and both implicated sex as an important predictor of MR’s central and cognitive benefits [17,19]. Their inclusion in these studies may be attributed to the increased susceptibility of females to age-related conditions [87], and the overwhelming disproportion of women who are affected by dementia and AD, comprising approximately two-thirds of all Alzheimer’s individuals [88,89]. Out of these two, one study included an in-depth investigation of MR’s efficacy on female working memory using the Y-maze [17]. This also happens to be the only study to have examined NORT and BM performances in females [17]. Therefore, this one study is providing the majority of evidence supporting MR’s efficacy on working, recognition, and spatial reference learning and memory in aged females [17]. However, neither study investigated MR’s ability to attenuate anxiety in females. This is especially problematic, considering there is a higher prevalence of anxiety disorder and increased anxiety-related disability among females compared to males across the lifespan [90,91]. Hence, going forward, MR studies are needed to examine its effectiveness against diet- and obesity-induced anxiety in females. Furthermore, studies including females should take into consideration the inherent male bias in many of these cognitive tests and ensure their studies are well designed and sufficiently powered to examine sexually dimorphic behavioral responses [92,93].

In a similar fashion, there is limited examination of MR’s effects on the female brain. For example, in the first study using females, MR improved working memory and spatial memory in both sexes and improved the brain state of males [17]. That said, MR had no effect on neuroinflammation, oxidative stress, or antioxidant expression in female brains [17]. In fact, the only central improvements reported in these females was increased synaptic ultrastructure and decreased mitochondria abnormalities [17]. Whereas in the second study, MR significantly improved neuroinflammation, oxidative stress, and spatial cognition in a male rodent model of AD, but failed to provide any anti-inflammatory or antioxidant protection to females, reflected in their poor MWM performances [19]. Interestingly, they also reported MR-induced improvements in female synaptic structure and reduced mitochondria abnormalities [19]. Studies have shown that the gut microbiota is also sexually dimorphic [94], and a recent publication revealed that female microbiomes respond differently to MR [95]. That said, no MR study examining the microbiome as a target of MR’s neurocognitive benefits included females [18,23]. Therefore, another major gap in MR research is an analysis of the effects of MR on gut health, specifically microbiome function and diversity, and its influence on cognition in females.

Perhaps the most important gap still remaining in MR research is identifying how MR’s *in vivo* benefits can be translated to humans. It is not uncommon to use animal models to better understand the pathology of human disease, but this often leaves a gap in the translatability of research findings. The level of MR that could produce cognitive benefits in humans is unknown. Considering that methionine is an essential amino acid found in almost every food, reconstructing this highly restrictive diet in humans has been shown to be somewhat challenging [96,97], and in general is not sustainable. Therefore, more research is needed to elucidate the exact cellular mechanisms responsible for MR’s central and cognitive protection in order to develop a more sustainable long-term approach. Such evidence can be seen in MR studies identifying FGF21 as essential for its cognitive benefits [17]. However, clinical trials using FGF21 analogs show limited efficacy on metabolic health and have yet to be evaluated in relation to the brain or cognition, revealing that other novel mechanisms must be at play [98].

## 2. Conclusions

**Figure 2:**
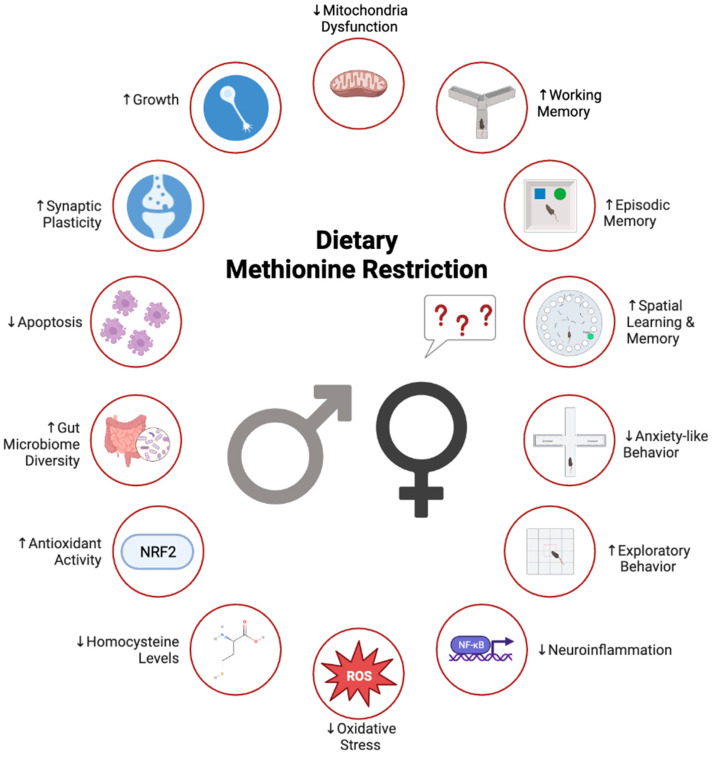
Overview of methionine restriction effects on cognition and potential mechanisms of action. MR improves cognition via improvements in overall brain health. However, sex disparities exist in these findings. Future research should also focus on better understanding the comprehensive effects of MR on female brain health and cognition. Created with Biorender.com.

Overall, MR has been shown to induce beneficial effects on cognition in various models including mouse models of aging, obesity, and AD. MR results in improvements in working memory, spatial memory, episodic memory, and anxiety-like behaviors. The ability for MR to improve learning and memory in tasks that depend on different brain regions, including the prefrontal cortex, amygdala, and hippocampus, suggests that MR induces a global protection for the brain, likely mediated by increases in FGF21, alterations in methionine metabolism pathways, reductions in neuroinflammation and central oxidative stress, and potentially alterations in the gut microbiome, mitochondrial function, and synaptic plasticity. Importantly, MR offers unique and powerful protection against metabolic dysfunction and cognitive decline without the need for caloric restriction. Interest in MR as a neurocognitive protectant in recent years has resulted in eight primary research publications, which are summarized in Table 1. However, these findings are still somewhat limited, and outstanding questions remain in our understanding of its comprehensive effects on the brain and cognition, especially in the female sex (Figure 2).

## Figures and Tables

**Table 1:** Methionine restriction research publications examining cognition.

Reference	Model	Diets	Cognition	Central Mechanisms
[19]	Male and female 4-month-old double transgenic APPswe/PSEN1dE9 mice (APP/PS1) with B6C3-Tg background and wildtype littermates (WT)	Standard chow (SD, *n* = 11–12, 0.86% Met) or Methionine restriction (MR, *n* = 11–12, 0.17% Met) for 16 weeks	MR improved the spatial cognition of males with AD, but impaired the spatial memory of WT males.	MR alleviated oxidative stress, increased mitochondria number and function, improved synaptic ultrastructure, and decreased amyloid-β in males with AD via the CBS/H_2_S pathway.
[17]	Study 1. Male C57Bl/6J mice aged 5- 15- and 18-months Study 2. Male 5- and 18-month-old mice injected with AAV-shFGF21 to knockdown *Fgf21* or AAV-scramble-shRNA as a control Study 3. Male and female 18-month-old C57BL/6J mice	Study 1. Control methionine (Con, *n* = 10, 0.86% Met) or Methionine restriction (MR, *n* = 10, 0.17% Met) for 12 weeks Study 2. Identical diets with and without *Fgf21* knockdown and (*n* = 8/group) for 12 weeks Treatment dose: AAV-shFGF21 (5 × 109 p.f.u. viruses per mouse via tail vein injection Study 3. Identical diets (*n* = 8/group) for 12 weeks	Study 1 Behavior: MR ameliorated aged-related decline in exploration, working, recognition, and spatial cognition in aged males. Study 2 Behavior: Endogenous FGF21 was essential for MR’s cognitive benefits on age-related decreases in working, recognition, and spatial cognition. Study 3 Behavior: MR improved working and spatial cognition of aged males and females.	Study 1: MR increased FGF21, improved synaptic ultrastructure, mitochondria biogenesis, and reduced inflammation and oxidative stress in the hippocampus of aged males. Study 2: Endogenous FGF21 was required for the neuroprotective effects of MR in aged males. Study 3: MR was equally effective at increasing FGF21, improving synaptic structure, and mitochondrial function in aged males and females.
[20]	Male 5-week-old C57BL/6J mice	Control (Con, *n* = 20, 4.2% fat, 0.86% Met) or High fat (HFD, *n* = 60, 24% fat, 0.86% Met) for 10 weeks to induce obesity Controls continued on Con for 16 weeks. 40 obese mice on the HFD were split into two groups: High fat (HFD, *n* = 20, 24% fat, 0.86% Met) or HFD + methionine restriction (HFD-MR, *n* = 20, 24% fat, 0.17% Met) for 16 weeks	MR reversed or normalized obesity-induced anxiety-like behaviors, recognition, and spatial learning and memory of obese mice.	MR increased H_2_S production, decreased circulating, and central inflammation, decreased oxidative species, and increased gene markers associated with synaptic function, neural plasticity, development, growth, and survival.
[23]	Male 10-week-old C57BL/6J mice	Standard chow (LFD, *n* = 84, 10% fat) or High fat (HFD, *n* = 84, 60% fat) for 4 weeks to induce obesity Each group then split into two groups: Standard chow + full methionine (LFD, group, *n* = 42, 10% fat, 0.86% Met) or Standard chow + methionine restriction (LFD+MR, *n* = 42, 10% fat, 0.17% Met) or High fat + full methionine (HFD, *n* = 42, 60% fat, 0.86% Met) or High fat + methionine restriction (HFD-MR, *n* = 42, 60% fat, 0.17% Met) for 8 weeks	MR improved HFD-induced cognitive deficits in working and spatial learning and memory.	MR improved HFD-induced alterations in neurotrophic factor expression, attenuated synaptic dysfunction in the hippocampus, improved gut function, inflammation status, and diversity.
[3]	Male 8-month-old C57BL/6J mice	Control (Con, *n* = 8, 0.86% Met + 4.2% fat) or High fat (HFD, *n* = 8, 0.86% Met + 24% fat) or HFD + methionine restriction (HF-MR, *n* = 8, 0.17% Met + 24% fat) for 25 weeks	MR restored HFD-induced cognitive deficits in recognition and spatial cognition of middle-aged male mice.	MR improved peripheral insulin sensitivity, increased insulin-related gene expression, increased antioxidant gene expression, and decreased ROS and other markers of oxidative stress, apoptosis, and H19 in the hippocampus.
[18]	Male 9-week-old ICR mice	Saline + Control (Con, *n* = 15, 0.86% Met) or D-galactose + Control (D+Con, *n* = 15, 0.86% Met) or D-galactose + Methionine restriction (D+MR, *n* = 15, 0.17% Met) Treatment dose: D-galactose 150 mg kg^−1^ day^−1^ for 8 weeks	MR improved d-Gal-induced anxiety, recognition, working, and spatial cognition in aging males.	MR decreased circulating and central inflammation and oxidative stress, increased brain, and body antioxidant activity, via increased H_2_S, restored genes involved in neural growth, survival, and synaptic plasticity, and improved microbiome diversity.
[22]	Male 9-week-old ICR	Low methionine (LM, *n* = 15, 0.17% Met) or Normal methionine (NM, *n* = 15, 0.86% Met) or High methionine (HM, *n* = 15, 2.58% Met) for 11 weeks	LM and NM improved anxiety-like behaviors, recognition memory, and spatial memory compared to HM-fed mice. However, LM and NM produced comparable beneficial effects on cognition.	Compared to HM, both LM and NM increased FGF21, microbiome diversity, short-chain fatty acid producing bacteria, serotonin, and antioxidants, and decreased central inflammation, oxidative stress, and gene markers of apoptosis.
[11]	Male 8-month-old C57BL/6J mice	Control (Con+MR0, *n* = 12, 0.86% Met + 4% fat) or Control + MR60% (Con+MR60, *n* = 12, 0.34% Met + 4% fat) or Control + MR80% (Con+MR80, *n* = 12, 0.17% Met + 4% fat) or Medium-fat control (MF-MR0, *n* = 12, 0.86% Met + 12% fat) or Medium-fat + MR60% (MF-MR60, *n* = 12, 0.34% Met + 12% fat) or Medium-fat + MR80% (MF-MR80, *n* = 12, 0.17% Met + 12% fat) or High-fat control (HF-MR0, *n* = 12, 0.86% Met + 22% fat) or High-fat + MR60% (HF-MR60, *n* = 12, 0.34% Met + 22% fat) or High-fat + MR80% (HF-MR80, *n* = 12, 0.17% Met + 22% fat) for 16 weeks	MR improved motor, anxiety, exploration, recognition, and spatial cognition, in a MR-dose and fat-dependent manner; 80% MR was the most effective at preventing all the HFD-induced impairments.	MR attenuated fat-induced neuroinflammation and oxidative stress, increased antioxidants and serotonin, decreased amyloid-β, increased neurogenesis genes in the hippocampus, improved thyroid function in a dose-dependent manner; 80% MR was the most consistently and effectively at protecting against varying fat intake.

## Data Availability

Not applicable.
